# Changing Ecotypes of Dengue Virus 2 Serotype in Nigeria and the Emergence of Cosmopolitan and Asian I Lineages, 1966–2019

**DOI:** 10.3390/vaccines11030547

**Published:** 2023-02-25

**Authors:** Bernard A. Onoja, Mamoudou Maiga, Ridwan O. Adesola, Andrew M. Adamu, Oyelola A. Adegboye

**Affiliations:** 1 Department of Virology, Faculty of Basic Medical Sciences, College of Medicine, University of Ibadan, Ibadan 200005, Nigeria; 2 Centre for Innovation in Global Health Technologies, Northwestern University, Chicago, IL 60611, USA; 3 Department of Veterinary Medicine, Faculty of Veterinary Medicine, University of Ibadan, Ibadan 200005, Nigeria; 4 Australian Institute of Tropical Health and Medicine, Building 48, James Cook University, Townsville, QLD 4811, Australia; 5 College of Public Health, Medical and Veterinary Sciences, James Cook University, Townsville, QLD 4811, Australia; 6 Department of Veterinary Public Health and Preventive Medicine, University of Abuja, Abuja 900105, Nigeria; 7 Public Health and Tropical Medicine, College of Public Health, Medical and Veterinary Sciences, James Cook University, Townsville, QLD 4811, Australia; 8 World Health Organization Collaborating Center for Vector-Borne and Neglected Tropical Diseases, College of Public Health, Medical and Veterinary Sciences, James Cook University, Townsville, QLD 4811, Australia

**Keywords:** dengue virus, ecotypes, sylvatic, Asian I, cosmopolitan strain, Nigeria

## Abstract

Dengue virus (DENV) is a leading mosquito-borne virus with a wide geographical spread and a major public health concern. DENV serotype 1 (DENV-1) and serotype 2 (DENV-2) were first reported in Africa in 1964 in Ibadan, Nigeria. Although the burden of dengue is unknown in many African countries, DENV-2 is responsible for major epidemics. In this study, we investigated the activities of DENV-2 to determine the circulating strains and to appraise the changing dynamics in the epidemiology of the virus in Nigeria. Nineteen DENV-2 sequences from 1966–2019 in Nigeria were retrieved from the GenBank of the National Center of Biotechnology Information (NCBI). A DENV genotyping tool was used to identify the specific genotypes. The evolutionary history procedure was performed on 54 DENV-2 sequences using MEGA 7. There is a deviation from Sylvatic DENV-2 to other genotypes in Nigeria. In 2019, the Asian I genotype of DENV-2 was predominant in southern Edo State, located in the tropical rainforest region, with the first report of the DENV-2 Cosmopolitan strain. We confirmed the circulation of other non-assigned genotypes of DENV-2 in Nigeria. Collectively, this shows that DENV-2 dynamics have changed from Sylvatic transmission reported in the 1960s with the identification of the Cosmopolitan strain and Asian lineages. Sustained surveillance, including vectorial studies, is required to fully establish the trend and determine the role of these vectors.

## 1. Introduction

Dengue is one of the most prevalent mosquito-borne viral infections and has emerged as a global public health problem [1,2]. Over 3.5 billion people in tropical and subtropical regions of more than 100 countries are at risk of dengue [3,4,5]. The disease is caused by any one of four closely related dengue virus (DENV) serotypes, DENV-1, DENV-2, DENV-3, and DENV-4, which belong to the family Flaviviridae, genus Flavivirus. Recent reports indicate that there is a fifth serotype [6], with each of the serotypes further subdivided into genotypes based on phylogenetic analysis. DENV is a positive-stranded ribonucleic acid (RNA) virus with a lipid envelope. The genome size is about 11 kb, with just one open reading frame (ORF) that is flanked by untranslated sequences at the 5′ and 3′ regions [7]. The ORF encodes one polyprotein that is divided into three structural (Capsid: C, Membrane: M, and Envelope: E) and seven non-structural proteins (NS1, NS2A, NS2B, NS3, NS4A, NS4B, and NS5). There are eight genotypes, namely Ia, Ib, II, III, IV, V, Recombinant, and Laboratory strains for DENV-1 and DENV-2. The latter is further subdivided into American, Asian-America, Asia I, Asia II, Cosmopolitan, Sylvatic, Recombinant, and unknown genotypes. DENV-3 has seven genotypes: I, II, III, IV, V, Recombinant, and unknown, while DENV-4 has six genotypes I, IIa, IIb, III, Sylvatic, and Recombinant [7]. The envelope protein facilitates infection through receptors as the virus interacts with the host cell. The E gene has shown high mutation rates, which can prevent neutralizing antibody activity [8].

The 1997 WHO case definition for dengue emphasized that clinical manifestations ranged from asymptomatic infection or mild dengue fever to dengue hemorrhagic fever (DHF) and dengue shock syndrome (DSS) [9]. This case definition was revised in 2009 to dengue and severe dengue [10]. About 390 million people in 100 countries are estimated to be infected with dengue annually [3]. DHF causes over 30,000 deaths, especially in the Asian-Pacific and American-Caribbean regions [11,12]. In recent times, DHF has been reported to be emerging on the West African front [13]. *Aedes species* are the vectors for dengue in Africa. *Aedes aegypti* is the primary vector, while *Ae. Albopictus* causes milder infections [14,15]. Both vectors are abundant in the tropical rainforest region of West Africa [16]. They spread as a result of human activity, such as international trade in used tires and mosquito-breeding in water collected in tree holes, plant leaf axils, bamboo stumps, rock pools, tins, cans, water storage containers, coconut shells, broken earthen and hollow ceramics, as well as water basins near peri-domestic habitation [17]. *Aedes species* are expanding their geographic space and appear unabated by several vector control programs in many parts of Africa [10]. Due to increased rural-urban migration, unplanned urbanization, population growth, and pesticide resistance of these vectors, dengue continues to spread without effective antivirals. The diversity of DENV genotypes and serotypes hampers the development of effective tetravalent vaccines. Additionally, intra-serotype genetic diversity in DENV results from recombination [18].

Since the nineteenth century, there have been reports of dengue in 34 African countries, with the DENV-2 serotype being responsible for most epidemics [19]. The DENV-2 Cosmopolitan genotype is the most common in Africa. It is estimated that 16% of the annual global burden is in sub-Saharan Africa, but the epidemiology is underreported in Africa [20]. The endemicity on the continent can be attributed to the abundant presence of the *Aedes species*, limited access to diagnostic facilities that can quickly identify ill patients, lack of standard healthcare facilities and awareness of the inherent danger of this neglected tropical disease, and lack of standard treatment [21]. In the past five decades, dengue outbreaks or sporadic infections have been reported in many countries in sub-Saharan Africa [10], including reports among travelers visiting West Africa [22].

In Africa, DENV was first isolated from humans at the Virus Research Laboratory Ibadan (now Department of Virology, Ibadan) in 1964 [23]. These included DENV-2 isolates belonging to the sylvatic lineage [24]. Dengue continues to be misclassified in many parts of Africa because of similar clinical signs and symptoms with malaria and other arboviral conditions. A study conducted in some urban parts of the rainforest region of Nigeria reported a rate of 23.4% active dengue, with high rates in April and August indicating efficient dengue transmission to humans [25], hence the need for effective dengue diagnosis. However, routine screening is not carried out because of ill-equipped laboratories and poor funding for surveillance. Recent dengue infections in Nigeria have identified DENV-2 as predominant [26]. Therefore, in the study, we investigated active dengue infection transmission patterns and described the genotypes and ecotypes of the circulating DENV-2 strains in Nigeria.

## 2. Materials and Methods

### 2.1. Data Sources

The FASTA format of DENV-2 sequences submitted from 1966–2019 in Nigeria was retrieved from the GenBank of the National Center of Biotechnology Information (www.ncbi.nlm.nih.gov) on 14 February 2023. They were all used in the phylogenetic and computational analysis.

### 2.2. Evolutionary History of DENV-2 Serotypes

A phylogenetic tree was constructed by comparing 17 DENV-2 sequences from Nigeria with 37 reference sequences deposited in the GenBank from Asia, Africa, Europe, and Australia. These reference sequences were randomly selected from DENV-2 serotypes after the BLAST search on the NCBI, representing each group of the submitted isolates. The New Guinea C prototype reference strain for DENV-2 detected in 1944 was included in the analysis. Multiple sequence alignment was performed with the retrieved DENV-2 sequences. The evolutionary history was inferred by using the maximum likelihood method based on the Tamura 3-parameter model [27]. The tree with the highest log likelihood (-17064.51) was shown. The percentage of trees in which the associated taxa clustered was shown next to the branches. Initial tree(s) for the heuristic search were obtained automatically by applying Neighbor-Join and BioNJ algorithms to a matrix of pairwise distances estimated using the maximum composite likelihood (MCL) approach and then selecting the topology with superior log likelihood value. The tree was drawn to scale, with branch lengths measured in the number of substitutions per site. The analysis involved 54 nucleotide sequences. Codon positions included were 1st + 2nd + 3rd + Noncoding. There were a total of 2354 positions in the final dataset. Evolutionary analyses were conducted in MEGA7 [28] and rooted with branch lengths in the same unit as those of the evolutionary distances. A DENV genotyping tool was used to identify the specific serotype and genotype available at https://www.genomedetective.com/app/typingtool/dengue/ (accessed on 13 February 2023).

### 2.3. Computational Analysis

DENV-2 Asian and Cosmopolitan lineages with query coverage of 100% and 99–100% identity were selected following a BLAST search in the GenBank, which included the Mahidol PDK95 vaccine strain in the DENV-2 Asian lineage and DENV-2 New Guinea C prototype. Sylvatic strains with query coverage of 100% and 82–100% identity were selected. BioEdit Sequence Alignment Editor version 7.2.6.1 was used to assemble and edit the DNA sequences [29]. Subtypes and genetic origins of the isolates were searched in GenBank for related or identical reference sequences using BLAST (http://blast.ncbi.nlm.nih.gov/Blast.cgi, accessed on 13 February 2023). The expasy tool from the Swiss Bioinformatics Resource Portal was used to translate all nucleotides from the isolate and reference sequences (https://www.expasy.org/, accessed on 13 February 2023). CLC Main Workbench 8.1 (QIAGEN, Aarhus A/S) was used to establish multiple sequence alignments of sequences from Nigeria and others received from GenBank. The software was further used to identify the amino acid mutations in the isolates.

## 3. Results

### 3.1. Prevalence of Dengue Virus (DENV) and (DENV-2) Isolates Characterized in Nigeria

**Figure 1:**
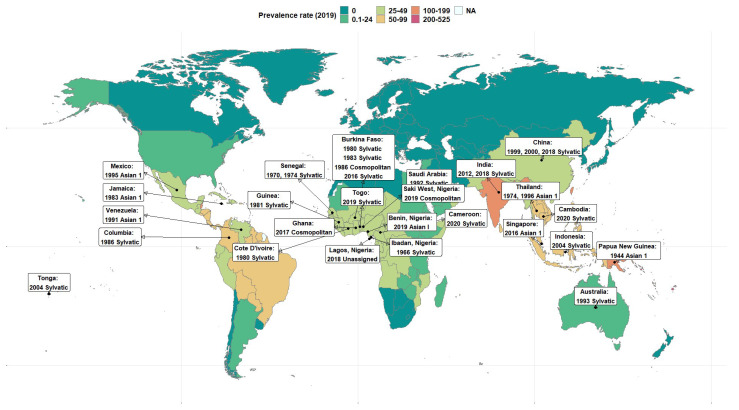
Map showing the global prevalence rate of dengue in 2019 and DENV-2 isolates. Source of mapped prevalence data: Global Burden of Disease Collaborative Network. Global Burden of Disease Study 2019 (GBD 2019) Results. Seattle, United States: Institute for Health Metrics and Evaluation (IHME), 2020. Available from https://vizhub.healthdata.org/gbd-results/ accessed on 13 February 2023. All rates are expressed as age-standardized based on the GBD reference population.

Nineteen DENV-2 isolates in Nigeria were deposited in the GenBank between 1966 to 2019 (Table 1). As far back as 1966, Sylvatic DENV-2 strains were identified in the human population. In 2018, there was a shift, although the specific genotype could not be assigned because the region of the virus that was identified was not sufficient for genotyping. In 2019, the first DENV-2 Cosmopolitan strain was reported in Saki West, Oyo State, where dengue had not been previously documented. In 2019, a different twist was observed in dengue epidemiology with the identification of the first Asian I genotype V in Edo State, located in the southern part of Nigeria (Table 1). Figure 1 presents the global geographical distribution of the burden of DENV and DENV-2 serotypes in this study. Supplementary File S1 contains a list of other DENV-2 serotypes from around the world.

### 3.2. Phylogenetics of DENV-2 Isolates in Nigeria and across the World

**Figure 2:**
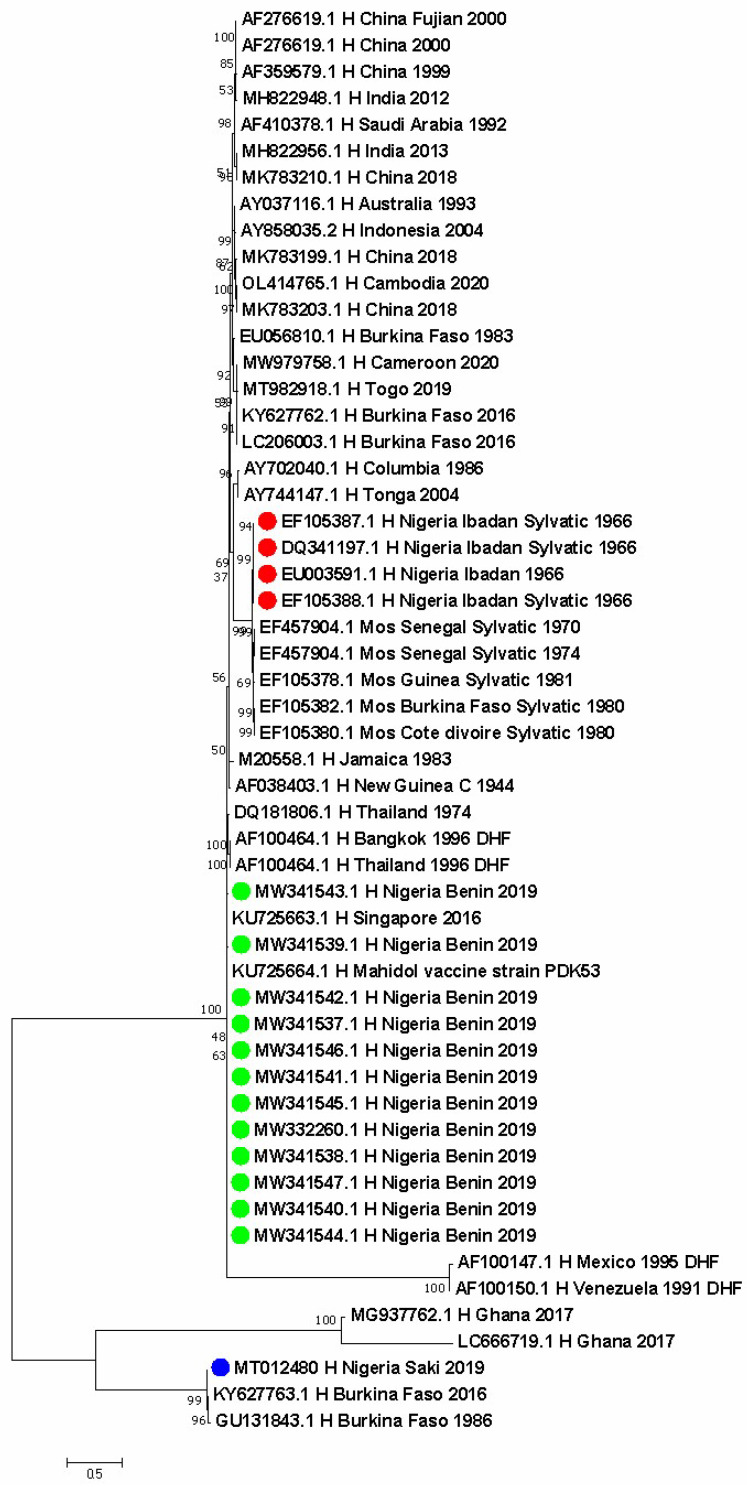
Evolutionary relationships of DENV-2 isolates from Nigeria compared with strains from other parts of the world. Dots are the specific locations in Nigeria. Red dot is the genotype VI Sylvatic strain (Ibadan, Oyo State), green is the genotype V Asian I (Benin, Edo State), and blue is the genotype II Cosmopolitan strain (Saki, Oyo State).

The evolutionary relationship of 54 DENV-2 isolates from different vegetation zones in Nigeria and other geographical regions, such as India, Australia, Saudi Arabia, Cameroon, Vietnam, Taiwan, Indonesia, Togo, Burkina Faso, Ghana, Senegal, Guinea, Cote D’Ivoire, India, Tonga, Columbia, Venezuela, Mexico, Thailand, Jamaica, Papua New Guinea, and China, are displayed in Figure 2. Nigerian strains identified in red are the four sylvatic DENV isolates from humans during the dengue epidemic in 1966. They show a close evolutionary relationship to sylvatic strains obtained from the Aedes species in Senegal in 1970 and 1974, in Cote d’Ivoire and Burkina Faso in 1980, and in Guinea in 1981. They also show a close relationship with strains that caused dengue hemorrhagic fever in Venezuela in 1991, in Mexico in 1995, and in Thailand in 1996. Further, they show a close association with strains from Togo in 2019 and Cameroon in 2020 (both countries share land borders with Nigeria). The DENV-2 Asian I genotype V strains in green detected in Nigeria are closely related to strains identified in Thailand in 1974 and 1996. The DENV-2 Cosmopolitan genotype VI identified in blue dots obtained from humans in Nigeria in 2019 is closely related to similar strains reported in two people from Burkina Faso in 2016 and in Ghanaians in 2017 (Figure 2).

### 3.3. Identification of Mutations in the DENV-2 Isolates

**Figure 3:**
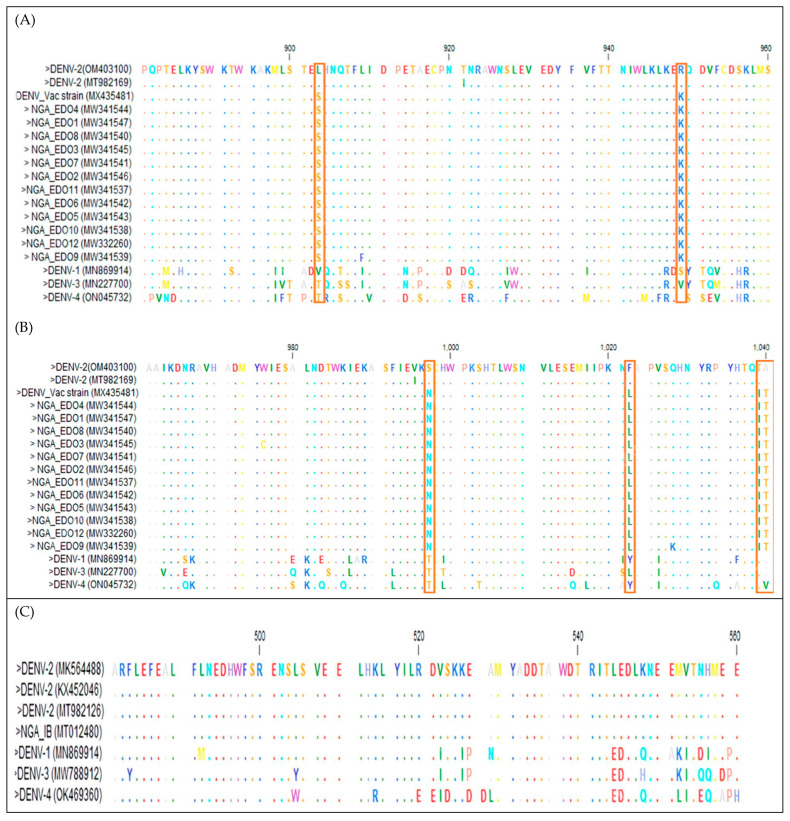

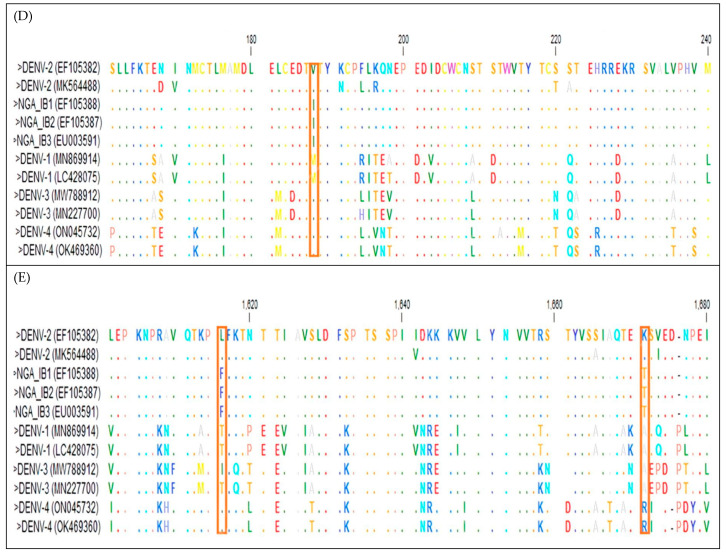
Multiple sequence alignment of the nucleotide sequences of (**top panel**, (**A**,**B**)) NS1, (**middle panel**, (**C**)) E/NSI, and (**bottom panel**, (**D,E**)) POLY genes of DENV-2. The highlighting indicates the position where amino acid mutations occurred in the genes of Nigerian isolates. Dots indicate positions where the gene sequences were identical to that of the consensus sequence.

A multiple sequence alignment (MSA) was performed with the NS1, E/NS1, and POLY genes of DENV-2, as shown in Figure 3. Despite the close relationship of the NS1, E/NS1, and Polyprotein with sequences from other countries, there were significant mutations, as shown in the pink box.

The MSA of the amino acid sequences of the NS1 protein of DENV-2 Asian lineage I sequence in Nigeria with others from the GenBank highlighted mutations in specific positions (903, 949, 997, 1022, 1039, and 1040) where amino acid mutations occurred in the NS1 proteins of Nigerian isolates and DENV vaccine protein (top panel, Figure 3A,B).

The MSA of the amino acid sequences of the E/NS1 protein of Cosmopolitan DENV-2 in Nigeria isolate indicated no highlighted positions in the sequences, meaning there was no amino acid mutation among the sequences. The MSA amino acid sequences of the POLY protein of Sylvatic DENV-2 sequences revealed significant mutations in specific positions (188, 388, 1370, 1532, 1615, 1671, 1897, 2658, 2902, and 3254) where amino acid mutations occurred in the POLY proteins of Nigerian isolates (bottom panel, Figure 3D,E). All these substitutions appear to be peculiar to Nigerian DENV-2 only, probably owing to local differentiation.

## 4. Discussion

In this study, we identified human dengue infection resulting from the DENV-2 Cosmopolitan strain in Nigeria. There have been several reports of dengue in Africa, but only in the early 1960s was DENV detected in humans in West Africa. It was originally discovered in Ibadan, the southwestern part of Nigeria, in 1960 [23]. Humans, *Aedes albopictus,* and *Aedes aegypti* are actively involved in the maintenance of DENV in urban areas [30]. As shown in the study, in the early days of dengue reporting in Nigeria, sylvatic strains were identified because monkeys and *Aedes species* from the heavily forested parts of the old Oyo Empire participated in the maintenance of DENV in the sylvatic cycle [30]. Nigeria and Senegal are two countries where this transmission cycle has been reported in febrile patients in West Africa [23,31]. In recent times, sylvatic dengue has not been linked to dengue fever in Nigeria; however, there are continuous reports in Senegal [32,33]. Although only Sylvatic DENV-2 has been reported in Africa, all four DENV serotypes have been reported to be transmitted in the sylvatic cycle in southeast Asia [34,35]. Even though there have been sporadic reports of dengue outbreaks in Nigeria since 1960, it is likely that many other dengue outbreaks are unnoticed, undiagnosed, and hence unreported [36]. It has been shown that *Ae. aegypti subsp. formosus*, which is the ancestral progenitor of *Ae. aegypti subsp. aegypti*, plays a limited role in the Sylvatic DENV-2 transmission cycle in the forests of West Africa [37,38]. Hence, it is resistant to infection with Sylvatic-DENV [37,38], and this may be why it is not so much of an issue in Nigeria, since it was reported that *Ae. albopitcus* is now more preponderant in the country [16].

Similarly, rapid migration and a high mutation rate within genotypes of DENV-2 render it prone to constant evolutionary changes, with the possibility of the emergence of new lineages [18]. In this study, we highlighted the first human dengue infection resulting from the DENV-2 Cosmopolitan strain in Nigeria, and the detection of the Asian I lineage in Benin–Edo State for the first time provided evidence of changing dynamics in dengue epidemiology. The phylogenetic analysis revealed that they span different geographic ranges, thereby supporting the theory that vegetation and climate play a significant role in mosquito-population dynamics, which affects arboviral diseases in Nigeria [2] and many parts of sub-Saharan Africa. The changes in the genotype landscape from previously identified sylvatic strains in Ibadan, Oyo State, Nigeria, underpins the silent infection of the DENV-2 Cosmopolitan strain in Saki, Oyo State, given that the strain has not been reported either within the country or in the neighboring Republic of Benin, with which it shares a land border. The particular patient had not traveled outside the locality within two months prior to the infection, giving us grounds to suspect that the infection was possibly an autochthonous transmission. It is plausible that active infection by the DENV-2 Cosmopolitan strain is ongoing because it is the predominant serotype/genotype in Africa [39].

The DENV-2 isolate detected in this study is the first Cosmopolitan strain in Nigeria and represents the fourth DENV-2 serotype isolated in the country, highlighting a shift from sylvatic lineage, or the addition of a new strain. This is an exciting finding because it indicates that DENV-2 has adapted to humans and is possibly maintained in human–mosquito–human transmission cycles, which no longer depends on animal reservoirs. One reason for this occurrence is the changing agricultural patterns. In the early 1960s, vegetation cover was thick in many parts of Oyo State, and most of the cases were detected among visiting outpatients attending the University College Hospital from all over the state. It is a different scenario from what was obtained in the Saki West area, where the present Cosmopolitan strain was detected. Saki West is not an urban center, and deforestation has led to increased agricultural activities, changing the virus–host–vector dynamics. It had been previously observed that the separation of forest and human transmission cycles reflected exclusive adaptation to their hosts (or vectors) [40], which may be at play in this instance. In November 2016, two travelers returning from Ouagadougou in Burkina Faso, West Africa, were diagnosed with non-complicated dengue fever in Marseille, France: a 25-year-old woman and a 28-year-old woman [41]. One of the four sequences (KY627763) showed close relatedness to the isolate from Saki West in Nigeria, while another isolate (GU131843), showing close homology to our isolate in this study, was isolated from a patient in Burkina Faso in 1986. This finding implies that DENV-2 Cosmopolitan strain is circulating in West Africa. In East Africa, DENV-2 Cosmopolitan genotypes were identified in 2014 and 2015 in Kilifi, Kenya; in the Taita–Taveta county, Kenya, in 2016; and in Malindi, Kenya in 2017, indicating sustained circulation of the Cosmopolitan strain in Kenya [42,43,44]. DENV-2 has not been reported in many West African countries, such as the Republic of Benin, which borders Nigeria to the east (and is closely located near Saki), and Togo. However, in 2017 two DENV-2 Cosmopolitan strains were identified from febrile patients in Ghana [45], which clustered closely to the Cosmopolitan strain in this study. It is unknown if the infected individuals in this study had a history of traveling to Ghana or outside Nigeria. However, given the location of Saki West in the hinterland and the semi-rural nature of the location, we are inclined to believe that there is a local transmission of DENV-2 Cosmopolitan strain in Saki, West Nigeria. This is because there have not been recent reports of sylvatic strains in Ibadan, given its proximity to Saki. The role of mosquitoes in the transmission of dengue in Saki West should be investigated, as DENV serotypes have been reported in eight ecological zones in Nigeria [46], and recent studies identified DENV-3 in *Aedes species* in Benue State, North Central Nigeria [47].

Several Asian I lineages were identified from different people in the same location in 2019. This is unprecedented; however, it is not clear if the source of transmission is local or imported, as the strain was hitherto largely confined to southeast Asia and parts of Latin America. Given the number of people from whom it was detected, there is sufficient reason to believe that the DENV-2 Asian I lineage is circulating in the Edo State, located in the tropical rainforest with heavy vegetation cover with intense breeding of *Aedes species.* More surveillance activities should be instituted in the area. Mutations in DENV-2 sequences in Nigeria show that evolutionary changes are taking place, and these must be monitored wholly along the entire length of the genome. Full-length sequencing of isolates should be considered in the future to fully elucidate the evolutionary changes and how they affect antigenicity, virulence, and phenotypic manifestations. A sublevel cluster analysis of sylvatic strains confirmed the presence of two lineages within the sylvatic genotype. All the sylvatic strains isolated from African countries (such as Senegal, Burkina Faso, Cote d’Ivoire, and Nigeria) formed a single cluster called S1 lineage.

On the other hand, the other isolates from other parts of the world belong to the S2 lineage. An earlier study reported the genetically distinct nature of the sylvatic strains from Malaysia and Africa [48]. Apart from the difference in the geographic origin, the strains from S1 and S2 lineages were observed to have distinct hosts. The strains of S1 lineage were isolated from either the mosquito or human host, whereas the S2 isolate was isolated from a monkey-like host; hence, this genetic diversity within the sylvatic genotype could be attributed to geographic origin and the host environment [7].

The mutations S903L, K949R, N997S, L1022F, I1039T, and T1040A found in all the DENV-2 of Asian lineage I isolates for the NS1 protein and DEN vaccine protein indicate a unique use of DENV-2 Asian lineage I as a vaccine candidate in Nigeria when compared with other strains. This was in contrast to a study where the E protein was used for subunit vaccines for DENV serotypes 1 to 4 [49]. Further, non-synonymous mutations I188V, A388V, I1370T, F1532L, F1615L, T1671K, T1897I, I2658T, E2902K, and S3254N observed in the DENV-2 of Sylvatic lineage for POLY protein appeared to be peculiar to Nigerian DENV-2 only, owing to local differentiation. Further research is necessary to determine the potential impact of these substitutions. The absence of notifiable changes seen in the amino acid of E/NS1 protein in all the DENV-2 of the Cosmopolitan lineage indicates no mutation of this strain. This strain serves as a useful reference for the development of regional diagnostics and research projects aiming at comprehending DENV evolution and transmission in Nigeria.

We acknowledge the following limitations in this study. Only 17 of the 19 sequences from Nigeria retrieved from the GenBank and VIPR were used for the phylogenetic analysis; 2 sequences from Lagos, Nigeria, in 2018 could not be included in the analysis as they were unassigned using the genotyping tool. There is a likelihood that other genotypes and strains could be identified with enhanced testing and detection strategies in autochthonous transmission cycles. Additionally, the sequences analyzed from Nigeria were based on publicly available sequences from 1966, 2018, and 2019. Complete genome sequence data are available for DENV-2 strains across many parts of the world, but this is lacking in Nigeria and is another limitation observed in this study.

## 5. Conclusions

In summary, this study identified human dengue infection resulting from the DENV-2 Cosmopolitan strain in Nigeria that necessitated sustained molecular surveillance in Nigeria to fully understand the dynamics of dengue epidemiology. Serotypic and genomic surveillance of dengue is important for early detection, identification of new introductions of genotypes associated with severity or higher transmissibility, or monitoring the emergence of sylvatic strains and genotypes. It is important for patient management because some clinicians are not aware of the high incidence of dengue; hence, it is not listed as a major cause of fever among patients in many parts. With the lack of currently available therapeutics for the treatment of dengue, this study also reinforces the need for vector control as an effective prevention strategy. The study reinforces the One Health approach to effectively identify dengue, both in hospital settings and at the animal–human–vector interphase, and to efficiently reduce the *Aedes species* population to control the spread of DENV. Additionally, there is a need to establish and maintain a national monitoring system for DENV and other arboviruses and to retrain healthcare professionals to diagnose dengue promptly. There is also an urgent need to provide better funding for research activities on DENV for comprehensive interventions that will lead to sequencing efforts and for improved phylogenetic analysis that will inform discussions on host interaction and transmission cycles. The outcomes will enhance understanding of the global DENV 2 genotype landscape and indicate the strains to be used in vaccine research, as vaccine development should take into account sylvatic strains from Nigeria and parts of West Africa where they were historically reported and continue to reemerge.

## Figures and Tables

**Table 1:** List of dengue virus serotype 2 obtained in Nigeria from 1966–2019.

SN	Accession No.	Genotype	Strain	Year	Location	Vegetation	Source
1	EF105387.1	Genotype VI	Sylvatic	1966	Ibadan, Nigeria	Derived savannah	Human
2	DQ341197.1	Genotype VI	Sylvatic	1966	Ibadan, Nigeria	Derived savannah	Human
3	EF105388.1	Genotype VI	Sylvatic	1966	Ibadan, Nigeria	Derived savannah	Human
4	EU003591.1	Genotype VI	Sylvatic	1966	Ibadan, Nigeria	Derived savannah	Human
5	MK045282.1 *	Unassigned	Unassigned	2018	Lagos, Nigeria	Mangrove, rainforest, and freshwater swamp	Human
6	MK045280.1 *	Unassigned	Unassigned	2018	Lagos, Nigeria	Mangrove, rainforest, and freshwater swamp	Human
7	MT012480.1	Genotype II	Cosmopolitan	2019	Saki West, Nigeria	Derived savannah	Human
8	MW341537.1	Genotype V	Asian I	2019	Benin, Nigeria	Rainforest	Human
9	MW341538.1	Genotype V	Asian I	2019	Benin, Nigeria	Rainforest	Human
10	MW341539.1	Genotype V	Asian I	2019	Benin, Nigeria	Rainforest	Human
11	MW341540.1	Genotype V	Asian I	2019	Benin, Nigeria	Rainforest	Human
12	MW341541.1	Genotype V	Asian I	2019	Benin, Nigeria	Rainforest	Human
13	MW341542.1	Genotype V	Asian I	2019	Benin, Nigeria	Rainforest	Human
14	MW341543.1	Genotype V	Asian I	2019	Benin, Nigeria	Rainforest	Human
15	MW341544.1	Genotype V	Asian I	2019	Benin, Nigeria	Rainforest	Human
16	MW341545.1	Genotype V	Asian I	2019	Benin, Nigeria	Rainforest	Human
17	MW341546.1	Genotype V	Asian I	2019	Benin, Nigeria	Rainforest	Human
18	MW341547.1	Genotype V	Asian I	2019	Benin, Nigeria	Rainforest	Human
19	MW332260.1	Genotype V	Asian I	2019	Benin, Nigeria	Rainforest	Human

* MK045282.1 and MK045280.1 could not be assigned to any genotype or strain; hence, they were not included in the evolutionary analysis.

## Data Availability

All sequence data used in this study are publicly available at the National Center for Biotechnology Information of the USA National Library of Medicine which is available at https://www.ncbi.nlm.nih.gov/ accessed on 13 February 2023.

## Supplementary Materials

The following supporting information can be downloaded at: https://www.mdpi.com/article/10.3390/vaccines11030547/s1, File S1: Dengue virus 2 isolates from across the different continents of the world.

## References

[1] Brady O.J., Gething P.W., Bhatt S., Messina J.P., Brownstein J.S., Hoen A.G., Moyes C.L., Farlow A.W., Scott T.W., Hay S.I. (2012). Refining the global spatial limits of dengue virus transmission by evidence-based consensus. PLoS Negl. Trop. Dis..

[2] Onoja A.B., Omatola C.A., Maiga M., Gadzama I.S. (2022). Recurrent Episodes of Some Mosquito-Borne Viral Diseases in Nigeria: A Systematic Review and Meta-Analysis. Pathogens.

[3] Yang X., Quam M.B., Zhang T., Sang S. (2021). Global burden for dengue and the evolving pattern in the past 30 years. J. Travel Med..

[4] Yamashita A., Sakamoto T., Sekizuka T., Kato K., Takasaki T., Kuroda M. (2016). DGV: Dengue Genographic Viewer. Front. Microbiol..

[5] Omatola C.A., Onoja A.B., Moses E., Mahmud M., Mofolorunsho C.K. (2021). Dengue in parts of the Guinea Savannah region of Nigeria and the risk of increased transmission. Int. Health.

[6] Mustafa M.S., Rasotgi V., Jain S., Gupta V. (2015). Discovery of fifth serotype of dengue virus (DENV-5): A new public health dilemma in dengue control. Med. J. Armed Forces India.

[7] Waman V.P., Kolekar P., Ramtirthkar M.R., Kale M.M., Kulkarni-Kale U. (2016). Analysis of genotype diversity and evolution of Dengue virus serotype 2 using complete genomes. PeerJ.

[8] Maginnis M.S. (2018). Virus-Receptor Interactions: The Key to Cellular Invasion. J. Mol. Biol..

[9] World Health Organization (WHO) (1997). Dengue Haemorrhagic Fever: Diagnosis, Treatment, Prevention and Control.

[10] World Health Organization (WHO) (2009). Dengue Guidelines for Diagnosis, Treatment, Prevention and Control.

[11] Mena Lora A.J., Fernandez J., Morales A., Soto Y., Feris-Iglesias J., Brito M.O. (2014). Disease severity and mortality caused by dengue in a Dominican pediatric population. Am. J. Trop. Med. Hyg..

[12] Jonduo M., Neave M.J., Javati S., Abala D., Bilo E., Kini A., Kumbu J., Laman M., Robinson L.J., Makita L. (2022). Genomic Sequencing of Dengue Virus Strains Associated with Papua New Guinean Outbreaks in 2016 Reveals Endemic Circulation of DENV-1 and DENV-2. Am. J. Trop. Med. Hyg..

[13] Fagbami A.H., Onoja A.B. (2018). Dengue Haemorrhagic Fever: An emerging disease in Nigeria, West Africa. J. Infect. Public Health.

[14] Gubler D.J. (2004). The changing epidemiology of yellow fever and dengue, 1900 to 2003. Comp Immunol. Microbiol. Infect. Dis..

[15] Ogunlade S.T., Meehan M.T., Adekunle A.I., Rojas D.P., Adegboye O.A., McBryde E.S. (2021). A review: Aedes-borne arboviral infections, controls and Wolbachia-based strategies. Vaccines.

[16] Onoja A.B., Adeniji J.A., Opayele A.V. (2016). Yellow fever vaccination in Nigeria: Focus on Oyo State. Highl. Med. Res. J..

[17] Irikannu K.C., Chukwuekezie O.C. (2015). Malaria and Man-Biting Mosquitoes in Tropical Africa; Omniscriptum.

[18] Chen R., Vasilakis N. (2011). Dengue—Quo tu et quo vadis?. Viruses.

[19] Amarasinghe A., Kuritsk J.N., Letson G.W., Margolis H.S. (2011). Dengue virus infection in Africa. Emerg. Infect. Dis..

[20] Yenamandra S.P., Koo C., Chiang S., Lim HS J., Yeo Z.Y., Ng L.C., Hapuarachchi H.C. (2021). Evolution, heterogeneity and global dispersal of cosmopolitan genotype of Dengue virus type 2. Sci. Rep..

[21] Were F. (2012). The dengue situation in Africa. Paediatr. Int. Child Health.

[22] Raut C.G., Rao N.M., Sinha D.P., Hanumaiah H., Manjunatha M.J. (2015). Chikungunya, Dengue, and Malaria Co-Infection after Travel to Nigeria, India. Emerg. Infect. Dis..

[23] Carey D.E., Causey O.R., Reddy S., Cooke A.R. (1971). Dengue viruses from febrile patients in Nigeria, 1964–1968. Lancet.

[24] Vasilakis N., Tesh R.B., Weaver S.C. (2008). Sylvatic Dengue Virus Type 2 Activity in Humans, Nigeria, 1966. Emerg. Infect. Dis..

[25] Onoja A.B., Adeniji J.A., Olaleye O.D. (2016). High rate of unrecognized dengue virus infection in parts of the rainforest region of Nigeria. Acta Tropica.

[26] Isa I., Ndams I.S., Aminu M., Chechet G., Dotzauer A., Simon A.Y. (2021). Genetic diversity of Dengue virus serotypes circulating among Aedes mosquitoes in selected regions of northeastern Nigeria. One Health.

[27] Tamura K. (1992). Estimation of the number of nucleotide substitutions when there are strong transition-transversion and G + C-content biases. Mol. Biol. Evol..

[28] Kumar S., Stecher G., Tamura K. (2016). MEGA7: Molecular Evolutionary Genetics Analysis version 7.0 for bigger datasets. Mol. Biol. Evol..

[29] Hall T.A. (1999). BioEdit: A user-friendly biological sequence alignment editor and analysis program for windows 95/98/NT. Nucleic Acids Symp. Ser..

[30] Fagbami A.H., Monath T.P., Fabiyi A. (1997). Dengue virus infections in Nigeria: A survey for antibodies in monkeys and humans. Trans. R. Soc. Trop. Med. Hyg..

[31] Robin Y., Cornet M., Heme G., Le Gonidec G. (1980). Isolement du virus de la dengue au Senegal. Ann. Virol..

[32] Diallo M., Ba Y., Sall A.A., Diop O.M., Ndione J.A., Mondo M., Girault L., Mathiot C. (2003). Amplification of the sylvatic cycle of dengue virus type 2, Senegal, 1999–2000: Entomologic findings and epidemiologic considerations. Emerg. Infect. Dis..

[33] Idrissa D., Niang S.S., Mignane N.M., Aliou B.M., Cheikh T., Moufid M., Diamilatou B. (2022). Detection of human case of dengue virus 2 belonging to sylvatic genotype during routine surveillance of fever in Senegal, Kolda 2021. Front. Virol..

[34] Rudnick A. (1986). Dengue virus ecology in Malaysia. Inst. Med. Res. Malays. Bull..

[35] Saluzzo J.F., Cornet M., Castagnet P., Rey C., Digoutte J.P. (1986). Isolation of dengue 2 and dengue 4 viruses from patients in Senegal. Trans. R. Soc. Trop. Med. Hyg..

[36] Otu A., Ebenso B., Etokidem A., Chukwuekezie O. (2019). Dengue fever—An update review and implications for Nigeria, and similar countries. Afr. Health Sci..

[37] Diallo M., Fernandez Z., Coffey L.L., Ba Y., Weaver S., Ortiz D., Mathiot C., Tesh R.B., Moncayo A.C., Sall A.A. (2005). Potential role of sylvatic and domestic African mosquito species in dengue emergence. Am. J. Trop. Med. Hyg..

[38] Diallo M., Ba Y., Faye O., Soumare M.L., Dia I., Sall A.A. (2008). Vector competence of Aedes aegypti populations from Senegal for sylvatic and epidemic dengue 2 virus isolated in West Africa. Trans. R. Soc. Trop. Med. Hyg..

[39] Langat S.K., Eyase F.L., Berry I.M., Nyunja A., Bulimo W., Owaka S., Ofula V., Limbaso S., Lutomiah J., Jarman R. (2020). Origin and evolution of dengue virus type 2 causing outbreaks in Kenya: Evidence of circulation of two cosmopolitan genotype lineages. Virus Evol..

[40] Monath T.P. (1994). Dengue: The risk to developed and developing countries. Proc. Nat. Acad. Sci. USA..

[41] Eldin C., Gautret P., Nougairede A., Sentis M., Ninove L., Saidani N., Million M., Brouqui P., Charrel R., Parola P. (2016). Identification of dengue type 2 virus in febrile travellers returning from Burkina Faso to France, related to an ongoing outbreak, October to November 2016. Euro. Surveill..

[42] Gathii K., Nyataya J.N., Mutai B.K., Awinda G., Waitumbi J.N. (2018). Complete Coding Sequences of Dengue Virus Type 2 Strains from Febrile Patients Seen in Malindi District Hospital, Kenya, during the 2017 Dengue Fever Outbreak. Genome Announc..

[43] Kamau E., Agoti C.N., Ngoi J.M., de Laurent Z.R., Gitonga J., Cotten M., Phan M.V.T., Nokes D.J., Delwart E., Sanders E. (2019). Complete Genome Sequences of Dengue Virus Type 2 Strains from Kilifi, Kenya. Microbiol. Res. Announc..

[44] Masika M.M., Korhonen E.M., Smura T., Uusitalo R., Vapalahti K., Mwaengo D., Jääskeläinen A.J., Anzala O., Vapalahti O., Huhtamo E. (2020). Detection of dengue virus type 2 of Indian origin in acute febrile patients in rural Kenya. PLoS Negl. Trop. Dis..

[45] Amoako N., Duodu S., Dennis F.E., Bonney J.H., Asante K.P., Ameh J., Mosi L., Hayashi T., Agbosu E.E., Pratt D. (2018). Detection of Dengue Virus among Children with Suspected Malaria, Accra, Ghana. Emerg. Infect. Dis..

[46] Baba M.M., Vorndam A.V., Adeniji J.A., Diop O., Olaleye D.O. (2009). Dengue Virus Infections in Patients Suspected of Malaria/Typhoid in Nigeria. J. Am. Sci..

[47] Agwu E.J., Isaak C., Igbinosa B.I. (2019). Detection of yellow fever and dengue viruses in mosquitoes between 2014 and 2015 in Bayelsa and Benue states of Nigeria. Acta Entomol. Serbica.

[48] Vasilakis N., Fokam E.B., Hanson C.T., Weinberg E., Sall A.A., Whitehead S.S., Hanley K.A., Weaver S.C. (2008). Genetic and phenotypic characterization of sylvatic dengue virus type 2 strains. Virology.

[49] Phan T.T.N., Hvasta M.G., Kudlacek S.T., Thiono D.J., Tripathy A., Nicely N.I., de Silva A.M., Kuhlman B. (2022). A conserved set of mutations for stabilizing soluble envelope protein dimers from dengue and Zika viruses to advance the development of subunit vaccines. J. Biol Chem..

