# Supplementary material for: Changing Ecotypes of Dengue Virus 2 Serotype in Nigeria and the Emergence of Cosmopolitan and Asian I Lineages, 1966–2019
Source: Vaccines (Basel). 2023 Feb 25;11(3):547. doi: 10.3390/vaccines11030547 (PMC10052944; doi:10.3390/vaccines11030547)
Supplement: Supplementary file 1 [file vaccines-11-00547-s001.zip › vaccines-2136057-supplementary.pdf]

Supplementary Table S1: Dengue virus 2 isolates from across the different continents of the world

SN	Serotype	Accession No.	Strain	Year	Location	Source
1	DENV-2	MT982918	Sylvatic	2019	Togo	Human
2	DENV-2	MW979758	Sylvatic	2020	Cameroon	Human
3	DENV-2	EF457904	Sylvatic	1970	Senegal	Aedes spp.
4	DENV-2	EF105382	Sylvatic	1980	Burkina Faso	Aedes spp.
5	DENV-2	EF457905	Sylvatic	1974	Senegal	Aedes spp.
6	DENV-2	AF276619	Sylvatic	2000	China	Human
7	DENV-2	AF359579	Sylvatic	1999	China	Human
8	DENV-2	EF105378	Sylvatic	1981	Guinea	Aedes spp.
9	DENV-2	EF105380	Sylvatic	1980	Cote D'ivoire	Aedes spp.
10	DENV-2	AY037116	Sylvatic	1993	Australia	Human
11	DENV-2	AF410378	Sylvatic	1992	Saudi Arabia	Human
12	DENV-2	AY858035	Sylvatic	2004	Indonesia	Human
13	DENV-2	KY627762	Sylvatic	2016	Burkina Faso	Human
14	DENV-2	AY702040	Sylvatic	1986	Columbia	Human
15	DENV-2	AY744147	Sylvatic	2004	Tonga	Human
16	DENV-2	EU056810	Sylvatic	1983	Burkina Faso	Human
17	DENV-2	LC206003	Sylvatic	2016	Burkina Faso	Human
18	DENV-2	AF276619	Sylvatic	2000	China	Human
19	DENV-2	OL414765	Sylvatic	2020	Cambodia	Human
20	DENV-2	MH822948	Sylvatic	2012	India	Human
21	DENV-2	MH822956	Sylvatic	2018	India	Human
22	DENV-2	MK783199	Sylvatic	2018	China	Human
23	DENV-2	MK783210	Sylvatic	2018	China	Human
24	DENV-2	MK783203	Sylvatic	2018	China	Human
25	DENV-2	AF038403	Asia 1	1944	Papua New Guinea	Human
26	DENV-2	M20558	Asia 1	1983	Jamaica	Human
27	DENV-2	AF100464	Asia 1	1996	Thailand	Human
28	DENV-2	KU725663	Asia 1	2016	Singapore	Human
29	DENV-2	KU725664	Asia 1	2016	Singapore	Human
30	DENV-2	AF100147	Asia 1	1995	Mexico	Human
31	DENV-2	DQ181806	Asia 1	1974	Thailand	Human
32	DENV-2	AF100464	Asia 1	1996	Thailand	Human
33	DENV-2	AF100150	Asia 1	1991	Venezuela	Human
34	DENV-2	LC666719	Cosmopolitan	2017	Ghana	Human
35	DENV-2	MG937762	Cosmopolitan	2017	Ghana	Human
36	DENV-2	GU131843	Cosmopolitan	1986	Burkina Faso	Human
37	DENV-2	KY627763	Cosmopolitan	2016	Burkina Faso	Human
